# Empirical analysis of the role of the environmental accountability system in energy conservation and emission reduction in China

**DOI:** 10.1038/s41598-022-19604-8

**Published:** 2022-09-10

**Authors:** Chunying Cui, Jing Li, Zhaoying Lu, Ziwei Yan

**Affiliations:** 1 Department of Economics and Management, Wenhua College, Wuhan, 430073 China; 2 Research Center of Internet and Industrial Innovation, Wenhua College, Wuhan, 430073 China; 3 School of Economics and Management, Beijing Forestry University, Beijing, 100083 China; 4 Adam Smith School of Economics and Finance, University of Glasgow, Glasgow, G12 8QQ UK

**Keywords:** Environmental economics, Energy conservation, Energy policy, Political economy of energy, Environmental social sciences, Energy economics

## Abstract

Many developing countries are facing the difficulty of choosing between economic growth and energy conservation and emission reduction (ECER). China has strengthened the implementation of ECER by setting environmental accountability as the development goal of local governments, hoping to have better governance effects. To evaluate the actual intervention effect of this approach, this paper constructs panel data covering 46 countries from 1995 to 2014 and uses the difference-in-differences (DID) method and the composite control method to quantitatively analyse the policy effect. The results show that China can effectively curb energy consumption and carbon emission intensity per unit of GDP by adding ECER targets to the government’s five-year plan, which has significant effects on ECER. Furthermore, we use an intermediary mechanism to test and identify low-carbon alternatives and an ECER promotion mechanism for technological advancement. The conclusion shows that economic development is compatible with low carbon and energy consumption. Combined with China’s long-term goals for ECER, it can be considered that on the road to achieving carbon peaking and carbon neutrality in the future, the economy and tertiary industry should be rationally developed, the degree of urbanization should receive more attention, and the proportion of thermal power generation should be reduced.

## Introduction

Global warming and the worsening of carbon emissions have seriously challenged the future situation of human survival and development. Since the beginning of the twenty-first century, the impact of climate change on human activities has become increasingly obvious, and major countries around the world are taking active measures to deal with these changes^1,2^. As the main producers of energy emissions in the current period, developing countries are different from developed economies. They face the contradiction between economic development and ECER constraints^3^. As the largest developing country, China has also become the economy with the largest carbon emissions due to its booming economy. Currently, it is actively responding to the above problems through its own efforts. Clarifying the main responsibilities of each local regime and including the ECER target in local governments’ economic development plan are an important measure that China has implemented for a long time to strengthen ECER work and achieve phased emission standards.

The characteristics of governance in China have mandated local regimes with strong authority, which is matched by competition to realize the target accountability system across the country^4^. In the 11th Five-Year Plan, the local regimes arranged the ECER target accountability system from the central government to replace the original GDP-oriented system, forming solid incentives for local bureaucrats and strengthening the implementation of existing ECER measures. This kind of target accountability system was included in the institutional innovation of government work objectives, which is obviously different from the existing ECER policy implementation mechanism in other countries around the world. Therefore, academics, policy-makers and environmentalists have paid much attention to the policy effects of this practice. However, the results of existing research on the policy effect of the ECER target accountability system are still very limited^5^, and no empirical study has analysed the policy effect of the ECER target responsibility system in China from the perspective of cross-country comparison. Therefore, this article employs cross-country counterfactual analysis to tackle this problem. The findings show that compared with other countries, China's unique policy accountability system for ECER has a remarkable effect on the enforcement of both energy conservation and emission reduction levels significantly after implementation. Thus, this effect can occur through the optimization of the energy power generation industrial structure.

China approved its accession to the Kyoto Protocol in 2008, clarifying its own ECER obligations and working with the international community to meet the enormous challenges posed by climate change. The Kyoto Protocol came into force in February 2005 as a supplementary clause to the United Nations Framework Convention on Climate Change (UNFCCC). As a developing country, China’s per capita carbon emissions are lower than the world average. Although there has been no restriction on absolute carbon emissions since its approval of the Kyoto Protocol, China has taken seven measures to address climate change issues through a series of ECER policies. However, the compliance level of these policies depends on the implementation of government responsibilities.

Before local governments’ ECER responsibilities were clarified, the government’s main responsibility for GDP growth, resulting in ECER compliance being limited to the environmental protection department’s responsibility as well as a lack of attention^6^. At that time, the government’s environmental regulation had the problem of soft policy constraints. Due to the accountability system based on GDP growth targets, local officials were obliged to ease environmental regulations to ensure economic growth. Based on the characteristics of China’s energy resources, the structure of energy use is still dominated by coal. In the historical period of the single GDP target accountability system, the consumption of fossil energies not only increases the agglomeration of local GDP but also causes environmental pollution, reducing the possibility of future sustainable development^7^. Therefore, many places have avoided ECER responsibility while pursuing economic growth.

In the Eleventh Five-Year Plan (2006–2010), China determined for the first time the binding indicators that governments at all levels need to achieve for ECER. Doing so was a major step forward in the implementation of ECER policy. The five-year plan is the economic development plan of the Chinese government. The government goals written into the five-year plan have become the focus of the work of government departments. Officials also rely on these goals to evaluate work performance. The policy practices of developing countries show that target accountability is an effective tool for local officials to achieve the central government’s policy goals^8^. However, regions outside of China have rarely implemented explicit ECER objectives through targeted accountability. This political uniqueness provides support for this paper and the subsequent selection of identification strategies.

In the Eleventh Five-Year Plan, the government’s economic development goals had to achieve ECER compliance. The compliance requirements for this emission reduction target were implemented in the "Notice of the State Council on Approving and Transmitting Implementation Plans and Measures for Energy-Saving and Emission Reduction Statistical Monitoring and Assessment" (hereinafter referred to as the "Notice") issued in 2007. The central government clearly stipulated that the completion of emission reduction targets would be regarded as an important basis for the comprehensive assessment of the performance of local governments and officials at all levels, and the accountability system for officials who did not meet the ECER standards and the "one-vote veto" system was implemented. The implementation of this "Notice" has greatly increased the ECER enthusiasm of officials everywhere, and many places have even relinquished some economic growth results to achieve emission reduction targets. To date, China has begun to form a scientific and systematic ECER effectiveness statistics and evaluation system, strengthening the role of pollution control compliance in officials’ performance and having a milestone effect on tightening pollution control. First, the Chinese government changed its overall development goals. From the single goal of realizing GDP growth to the dual goal of realizing the common development of the economy and the environment, economic development cannot occur at the expense of the environment. Environmental governance has become a hard constraint on growth. Economic growth is achieved under environmental constraints. The two are mutually tolerant. Second, environmental protection has become an obligation for the government as the main body through official accountability. The attribution of responsibility to achieve goals and standards^5^ has suppressed the deterioration of the environment unrestrainedly in various regions. If the local environment does not meet the corresponding goals, the promotion of local officials will also be restricted, aligning the orientation of officials’ personal interest and the public’s environmental interests, thus realizing the common development of the economy and the environment and changing the previous situation, which was oriented only towards economic development. This shift has realized the internalization of the externalities of environmental regulations. Third, the five-year plan is consistent. Consistency means that environmental protection policies will continue to play a role in the future for a long time.

Before incorporating ECER targets into the five-year plan, China had issued corresponding laws and regulations to restrict environmental development. As shown in Table 1, China had previously restricted environmental development mainly through administrative orders—control-led methods. Subsequently, the policy changed its regulatory means, turning to laws, regulations and economic means to achieve the goal of energy savings and emission reduction.

**Table 1:** Evolution of China’s ECER policies before 2006.

Time	Event
1979	Promulgation of "Environmental Protection Law (Trial)"
1992	"Ten Countermeasures for China’s Environment and Development"
1994	Announced "Agenda 21 of China"
1995	Determine "Implementing two fundamental changes"。
2002	Ratification of the "Kyoto Protocol"
2005	The “Kyoto Protocol” enters into force
2006	Accelerating the introduction of policies on ECER since the 11th Five-year Plan

The information collected by author.

For China, the five-year plan holds great significance. In the plan, binding indicators are set to promote the realization of energy saving and emission reduction targets. From the beginning of the Eleventh Five-Year Plan to the present, each successive Five-Year planning government has set specific binding targets.

After China’s accession to the World Trade Organization, the degree of openness to the outside world and the degree of trade further increased, resulting in the problem of carbon transfer. Approximately 20% of China’s domestic emissions are due to consumption in developed countries. Many developed countries have transferred their own emissions to trade partners from which they import goods^9^. The implied increase in energy consumption in exports is due to the expansion of the export scale^10,11^. Therefore, the coordination of the relationship between China’s economic development and environmental pollution also warrants attention. The Porter hypothesis^12^ states that ECER will not inhibit economic development; rather, appropriate environmental control policies will promote technological progress, thereby increasing the production capacity of enterprises. In China, the Chinese government can implement conservative energy policies and carbon emission policies without affecting economic growth^13^. The research of Wang et al.^14^ also supports the Porter hypothesis and argues that differentiated energy saving and emission reduction policies will further promote the green economy.

Most research results indicate that the energy structure, the industrial structure and urbanization have an important impact on the effectiveness of ECER^15–18^. However, the energy structure, trade openness and urbanization have a two-way causal relationship with carbon dioxide emissions^19^. The energy structure accounts for the largest proportion in terms of the factors that affect carbon emission intensity^20^. The regional equilibrium of emission intensity^21^ is followed by the influence of enterprise equilibrium^22,23^. China promises to peak its carbon emissions by 2030. Only when the constraint intensity of environmental governance policies gradually increases can energy structure optimization and carbon emission intensity be forced to ease, while the peak in fossil energy consumption and carbon emissions can jointly be achieved in advance^24^. The continuous promulgation of environmental legislation and environmental constraint policies attempts to strengthen such government administration pressure^25^, but the difference between the strength and weakness of policy implementation is ignored^26^.

Similar research has shown that, compared with the past period, China’s energy saving and emission reduction policies and plans formulated during the Eleventh Five-Year Plan period have made significant progress, and many plans that even exceeded their energy saving targets have been completed. However, with the deepening of policy implementation, during the 13th Five-Year Plan period, China’s economic development goals are easily achieved, but the level of development in various regions is uneven, causing most regions’ own economic goals and ECER goals to be mismatched, resulting in unsatisfactory energy saving and emission reduction effects^27^. Current studies focus on the fact that this enormous change has continued through the 13th Five-Year Plan period. At present, analysis based on government targeted accountability focuses on environmental protection^28,29^, and some studies have found differences in national development caused by environmental technology progress^30^, including the differences in growth caused by the adoption of renewable energy^31^, but a cross-country comparison of ECER effectiveness between China and other countries in the same period is lacking.

In summary, energy consumption and emissions are the focus of many studies on ECER, and there are enough research results to show the changing relationship between China’s economic development and ECER. There are also sufficient achievements to show the effectiveness of ECER-related policies in China^32^. However, there are limited studies evaluating the effectiveness of officials’ accountability for environmental constraint goals^33^. To better realize the sustainable development goals of the UN, this paper evaluates the innovation effect on institutional tools when the central government adopts a target accountability system to strengthen the intensity of ECER policy. If we regard China's local regime undertaking the target responsibility system of ECER in the five-year plan as a quasi-natural experiment, the effect of the government's policies on ECER can be evaluated from different aspects through two observation variables: energy consumption per unit GDP and carbon emission intensity. Based on these assumptions, this paper employs the DID method and the SCM to conduct counterfactual estimation. Compared with ordinary regression, the counterfactual estimation method can capture the impact of policy implementation in full probability events, which can avoid the overidentification of policy effects caused by other common factors, such as legislation on environmental protection and the signing of the Paris Climate Agreement. To ensure that the results are not accidental, we also use sensitivity analysis and permutation tests to prove that the estimation results are less sensitive and are robust.

Compared with existing similar studies, this paper makes the following marginal contributions: (1) Taking the accountability system of ECER targets as a variable affecting policy impacts, this paper empirically discusses the actual effect of local officials’ efforts to achieve ECER goals from the perspective of the government's accountability for the implementation of existing policies, creating a new dimension in research. (2) This paper conducts a horizontal comparison of ECER implementation effects across countries with a long time span, reflecting the implementation results of China's unique policy tools and adding new empirical evidence to recent ECER country studies. It differs from existing studies that focus on conducting a vertical comparison of ECER policy effects in China. (3) This research finds that the mechanism of the target accountability system implementing ECER policy is increasing the proportion of clean power generation, providing a new path explanation for exploring the transmission mechanism of ECER policy.

## Research hypotheses

The environmental accountability system for officials has been implemented since the Eleventh Five-Year Plan. The ECER target has been widely written into the work outline of local governments and has become one of the key guidelines in evaluating the performance of major officials. Previously, since local government economic development goals have long been set based on GDP growth, there was a "tournament" mechanism to compete for the rate of economic increase in China^4^. There was no corresponding mechanism for local governments to undertake their due obligations with regard to environmental protection and ECER problems. Thus, before 2012, there was statistical manipulation of ECER, which empirical evidence subsequently eliminated^34^. The enhancement of this promotion mechanism has been deeply understood, convincing people with experience of economic construction in the past 30 years. When the economic growth target gave way to the environmental target, institutional inertia formed a strong binding force on local governance. Local governments explicitly established ECER targets in the five-year plan to initiate "promotion tournament" competition under a new accountability orientation. Thus, ECER policies and regulations have been implemented effectively. In the successive five-year plans after the Eleventh Five-Year Plan, ECER objectives have been continuously promoted, forming a long-term consistency of environmental policy constraints, actively promoting the management of the demand side, and reducing emissions by improving energy efficiency^35^, leading to sustained ECER effects.

Therefore, this paper proposes Hypothesis 1: The environmental target accountability system implemented in the Eleventh Five-Year Plan reduces energy consumption per unit production. Furthermore, it proposes Hypothesis 2: The environmental target accountability system also reduces the intensity of carbon emissions.

The increase in the scale of carbon emissions comes directly from the energy consumption in the production process. Therefore, reducing carbon emission intensity depends on continuous ECER efforts, which technically reduce the energy consumption per unit of output value^36^. Energy consumption increases gradually with economic growth. Although the use of market mechanisms and institutional supply can continuously reduce the energy consumption per unit of output value to gradually decouple energy consumption from the process of economic growth^37^, the overall trend of total energy consumption expanding with economic output will not be reversed. Therefore, improving the primary energy structure and gradually replacing fossil energy with renewable energy and low-carbon energy has become a technical path for reducing carbon emission intensity^38^. All countries have made continuous efforts in regard to the energy structure of power generation, continuously reducing the proportion of fossil fuel power generation and increasing the installed proportion of renewable energy power generation to achieve cleaner production^39^. China's environmental accountability system has accelerated the pace of the coal-removal process in power generators by local governments. Due to the requirements of the carbon emission mitigation target of the five-year plan, local governments have actively fulfilled their environmental protection responsibilities, made efforts to adjust the structure of primary energy power generation, vigorously installed clean energy generating units such as hydropower, wind power and photovoltaic power generation, and shut down existing thermal power units or replaced them with natural gas low-carbon power generation^40^, which popularizes clean power generation technology by reducing the proportion of thermal power generation to effectively reduce carbon emission intensity^41^.

Therefore, this paper proposes Hypothesis 3: The environmental accountability system implemented in the Eleventh Five-Year Plan promotes a reduction in carbon emission intensity by reducing the proportion of thermal power generation.

## Methodology

### Variables and data

In this paper, the BP Energy Yearbook is selected to build a cross-country database of energy consumption and carbon emissions, and the data source of this study is the economic and social development data of the World Bank’s World Development Indicators (WDI). While the Chinese government is pushing for ECER accountability, other countries are not implementing similar policies. Therefore, causal inferences about the effects of environmental accountability policies rely on country comparisons between China and other countries that have not implemented environmental accountability policies, thus constituting counterfactual estimates. Based on the availability of data, this paper excluded the samples of economies with a large amount of long-term missing data, as well as individual samples with large significant differences. Ultimately, it recorded data on the energy consumption, carbon emissions and economic and social development of 46 countries, and these data constitute the research database of this paper.

By constructing panel data covering 46 countries from 1995 to 2014, this article explores the impact of China’s transformation of the policy adjustment method to achieve ECER targets on carbon emission intensity and energy consumption per unit of GDP. We try to limit the time node to 2014 because the ECER accountability system for county mayors was made stricter and upgraded to that for local communist party secretaries in 2015. In addition, policy shocks, such as “Article 10 on the atmosphere in northern provinces”, using gas instead of coal for winter heating, and the “low-carbon city pilot zone”, might lead to confusion and distort the real effect of the accountability policy effect. A series of new ECER-concerned policies was provided in China after 2015, and these environmental policies with Chinese characteristics modified the single target to multiple targets in regard to the accountability of local bureaucracies. The general rule revealed in this paper is that the policy effect brought by ECER accountability is significant. The latest literature has already illustrated similar applications by using the same period until 2014 for local official accountability studies in China^5,29,42^.

The key observation variables for the transformation of environmental regulation and adjustment methods are energy consumption and emissions. Energy consumption and carbon emission intensity per unit GDP are selected as outcome variables because they serve as the essential observation variables for the transformation of the mode of environmental regulation. In the environmental field, scholars often apply the IPAT model, proposed first by Ehrlich and Holdren^43^, who argued that population size, wealth and the technology level are the main factors affecting the environment. Due to the limitations of the IPAT model, Dietz and Rosa^44^ established the STIRPAT model on the basis of the IPAT model, and many scholars have also expanded the model to make it more suitable for their own research^45^. Therefore, this article follows the methods of previous scholars to select the control variables from the above-mentioned aspects.

Referring to previous studies, the control variable set of this paper is shown as follows: Urbanization leads to the aggregation of human activities, increasing the volume of energy consumption and carbon emissions. Therefore, the urban population is selected as the proxy variable for the country's population size^46^. Energy consumption and carbon emissions are directly affected by a country's economic aggregate; thus, gross domestic product (GDP) is used as a proxy variable^5^. The development level of the tertiary industry is adopted to reflect the characteristics of the industrial structure to optimize the industrial structure and reduce energy consumption and total carbon emissions^47^. The replacement of clean energy will effectively reduce carbon emissions^48^. In this paper, the proportion of clean power generation is used as a proxy variable for the optimization of the energy utilization structure. Except for ratio data, all other data are processed in logarithmic form. Table 2 shows the source of each variable.

**Table 2:** The source of each variables.

Variable	Description	Units	Sources
CI	Carbon intensity	Kg/PPP USD GDP	World Development Indicators (WDI)
EC	Energy consumption per unit of GDP	Standard coal ton consumption for oil, gas and coal/GDP	Standard coal ton consumption for oil, gas and coal from bp Statistical review of world energy; GDP from WDI
URB	Urban population	Person	World Development Indicators (WDI)
GDP	Economic level	Current dollar	World Development Indicators (WDI)
SER	The level of development of the tertiary industry	Service trade volume (% of GDP)	World Development Indicators (WDI)
CPG	Energy use structure	Thermal power generation (% of total)	World Development Indicators (WDI)

## Measurement model

### Difference-in-differences method

To analyse the effects of ECER after China’s transformation of environmental regulations, this paper adopts the DID method, which has been a commonly used approach for the causal inference of policy effects since David Card (1993) ^49^. In this paper, a quasi-natural experiment is formed by incorporating the ECER index into provincial government accountability as a policy impact variable in China's 11th Five-Year Plan in 2006. The net effect of the policy is evaluated by comparing the significant observed differences in ECER characteristics between China and other control countries after 2006. The specific model settings are as follows:

1 $$EC_{it} = \beta_{0} + \beta_{1} ECER_{it} \times Year_{it} + \beta_{2} ECER_{it} + \beta_{3} Year_{it} + \beta_{4} URB_{it} + \beta_{5} GDP_{it} + \beta_{6} SER_{it} + \mu_{it}$$

2 $$CI_{it} = \lambda_{0} + \lambda_{1} ECER_{it} \times Year_{it} + \lambda_{2} ECER_{it} + \lambda_{3} Year_{it} + \lambda_{4} URB_{it} + \lambda_{5} GDP_{it} + \lambda_{6} SER_{it} + \nu_{it}$$

By passing the parallel trend test, the horizontal changes in energy consumption and emissions for all sample groups and the vertical changes in China's temporal factors can be differentiated to show the impact of policy implementation. In Eqs. (1) and (2), and denote the urban population, economic level, tertiary industry development level, energy consumption level, and emission level, respectively. Dummy variables indicate whether the government has changed the adjustment mode of environmental regulations. If the country changes the adjustment mode, the value is 1; otherwise, it is 0. It is a time dummy variable. If the policy window period is before 2006, the value is 0; after 2006, the value is 1, the interaction term measures the policy effect, and both are random disturbance terms.

### Synthetic control method

However, the DID method also has limitations. First, it is easy to produce differences between the treatment group and the control group, and it is difficult to avoid policy endogeneity. Therefore, this paper further uses the synthetic control method for analysis and generates the weight of each control group through data driving, which reduces the possible selection bias and endogeneity problems of the control group and makes the conclusion more reliable.

The basic idea of the synthetic control method is that it fits other control groups with corresponding weights to fit the situation of the research object without policy. Then, it compares this situation with the actual situation to obtain the policy effect.

Suppose that there is a country. The first country is the intervention group, and the remaining countries are the control group; Then,

3 $$Y_{it}^{{}} = Y_{it}^{0} + \alpha_{it} D_{it}$$

where $Y_{it}^{{}}$ represents the value of the individual’s result variable $i$ observed at time $t$ and $D_{it}$ is a dummy variable. If policy intervention is carried out, the value is 0; otherwise, it is 1. Unknown $Y_{it}^{0}$ is an observation under the counterfactual framework, so it is necessary to estimate the volume of the net policy effect $\alpha_{it}$.

Driven by the data, a linear combination of different weights can be given to the control group to form a counterfactual result:

4 $$Y_{1t}^{0} { = }\delta_{t} { + }\theta_{t} Z_{i} + \lambda_{t} \mu_{i} + \varepsilon_{it}$$

Only by obtaining the $K \times 1$ dimensional weight vector $W^{*} = (w_{2} ,...,w_{N + 1} )$ can we obtain the observation value under the counterfactual framework:

5 $$\sum\limits_{k = 2}^{K + 1} {\omega_{{\text{n}}}^{{}} } Y_{it} { = }\delta_{t} { + }\theta_{t} \sum\limits_{k = 2}^{K + 1} {\omega_{k}^{{}} } Z_{k} + \lambda_{t} \sum\limits_{k = 2}^{K + 1} {\omega_{k}^{{}} } \mu_{k} + \sum\limits_{k = 2}^{K + 1} {\varepsilon_{kt} }$$

If there is a weight vector set $(w_{2}^{{}} ,...,w_{N + 1}^{{}} )^{\prime}$ that meets the condition of equation, then

6 $$\sum\limits_{k = 2}^{K + 1} {w_{k}^{*} Y_{k1}^{{}} = Y_{11}^{{}} } ,...,\sum\limits_{k = 2}^{K + 1} {w_{k}^{*} Y_{{kT_{0} }}^{{}} = Y_{{1T_{0} }}^{{}} } ,\quad \sum\limits_{k = 2}^{K + 1} {w_{k}^{*} Z_{k} } = Z_{1}$$

When $\sum\limits_{t = 1}^{{T_{0} }} {\lambda_{i}^{\prime } \lambda_{i} }$ is nonsingular, we can obtain the equation:

7 $$Y_{1t}^{0} - \sum\limits_{k = 2}^{K + 1} {w_{k}^{*} } Y_{kt}^{{}} = \sum\limits_{k = 2}^{K + 1} {w_{k}^{*} } \sum\limits_{s = 1}^{{T_{0} }} {\lambda_{t} } \left( {\sum\limits_{i = 1}^{{T_{0} }} {\lambda_{i}^{\prime } \lambda_{i} } } \right)^{ - 1} \lambda_{s}^{\prime } \left( {\varepsilon_{ks} - \varepsilon_{1s} } \right) - \sum\limits_{k = 2}^{\begin{subarray}{l} \\ K + 1 \end{subarray} } {w_{k}^{*} \left( {\varepsilon_{kt} - \varepsilon_{1t} } \right)}$$

Abadie^50^ proved that under normal circumstances, the above formula is close to 0, and $\sum\limits_{k = 2}^{K + 1} {w_{k}^{*} } Y_{kt}^{{}}$ can be used as an unbiased estimator. It is an unbiased estimator that obtains the size of the policy effect.

In this study, we choose the urbanization level, GDP scale, tertiary industry development level and primary energy structure as prediction variables to improve the fitting effect of the policy pre-period, and some period-lagged values of the outcome variables are added as the predictor variables. However, the zero weights are always occupied by employing all of the past values of the outcome variables as the predictor variables^51^.

## Results

### Variable description

Based on panel data covering 46 countries from 1995 to 2014, this paper studies the promoting effects of China’s environmental regulatory policies in 2006 on ECER target accountability. Table 3 shows the descriptive statistics of each variable. Except for EC and CPG, the standard deviations of the other variables are small. The skewness value shows that except for left-biased URB, the other variables are all right biased. The Jarque–Bera value indicates that all variables obey a normal distribution.

**Table 3:** Variable descriptive statistics.

	EC	CI	URB	GDP	SER	CPG
Mean	13.595	0.380	16.766	26.404	29.030	29.105
Median	6.847	0.314	16.843	26.271	13.337	23.288
Min	0.106	0.070	12.733	23.149	25.599	0
Max	154.696	2.191	20.422	30.495	32.457	94.770
SD	19.809	0.2603	1.425	1.455	1.389	25.925
Skewness	3.133	2.734	− 0.093	0.396	5.991	0.719
Kurtosis	15.125	13.451	3.329	2.821	44.314	2.464
Jarque–Bera	0	0	0.065	3.279e−06	0	2.546e−20
Observations	920	920	920	920	881	920

## Counterfactual estimation

### DID

Based on the model setting, the output of the DID method is shown in Table 4. In Model (1) and Model (3), only individual fixed effects and time fixed effects are controlled. In Model (2) and Model (4), the control variables *GDP*, *URB* and *SER* are added to represent the interaction term ECER*Year of the policy effects. TIME is significant at the 1% level, the explanatory power of the model has become stronger, the coefficient has also become larger, and the coefficient increases from − 0.192 to − 0.208. This result shows that the transformation of environmental regulatory policies has a positive impact on energy consumption per unit of GDP and a negative impact on carbon emission intensity. The study of Wang and Liu^14^ also shows that ECER policies can achieve a win‒win situation for the economy and the environment.

**Table 4:** Regression results of DID.

Variable	Energy consumption per unit GDP		Carbon emission intensity	
	Model(1)	Model(2)	Model(3)	Model(4)
ECER*YEAR	8.557** (3.455)	20.205*** (4.482)	− 0.192*** (0.047)	− 0.208*** (0.034)
*LNGDP*		− 28.192*** (6.158		− 0.191*** (0.043)
*LNURB*		38.749** (18.610)		0.812** (0.259)
*SER*		− 0.278*** (0.081)		− 0.002* (0.001)
Cons	19.894*** (4.745)	111.146 (232.335)	0.424*** (0.036)	− 8.232** (3.618)
R^2^	0.304	0.595	0.476	0.661
Obs	920	881	920	881

In parentheses are standard errors, *, ** and *** indicate significance levels of 1%, 5% and 10%, respectively.

Clearly, the regression result of energy consumption per unit of GDP is not in line with expectations. The main reason is that, compared with the synthetic control method, one of the shortcomings of the DID method is that it is difficult to separate the policy, and it may misidentify the result due to policy proliferation. We found that in December 2001, China joined the World Trade Organization and gradually formed the world factory development model, expanded the degree of openness to the outside world, exported a large number of industrial products to the world market, and achieved structural changes in production. Production energy consumption greatly increased, and the impact of joining the World Trade Organization on energy consumption per unit of GDP is greater than the impact of changing policy adjustment methods. Therefore, the regression results of energy consumption per unit of GDP using the DID method are inaccurate.

For carbon emission intensity, we have reason to use the DID method for estimation. The reason is as follows: Although China’s accession to the World Trade Organization led to structural changes in production, carbon emission intensity is mainly affected by a country’s technology level, and the contribution rate of technological energy savings can reach 56%^20^. Under the same production level, an improvement in technology can reduce the intensity of carbon emissions, and there is no structural change in the development of technology.

In summary, the regression results obtained by using the DID strategy show that energy saving and emission reduction policies have significant effects on emission reduction, while energy-saving aspects need to be further analysed. Therefore, Hypothesis 2 is validated, while Hypothesis 1 is not.

### Synthetic control method

(1) Energy consumption per unit of GDP

In the next stage, the SCM is adopted in this paper to further identify the policy effect after adding the ECER indicator to the accountability of local regimes. Through the calculation and output of Stata, using the predictive variable matrix, linear programming generates the weight vector matrix, thereby fitting China, which is unobserved. Among the 45 control groups, there are five countries in the control group with non-zero weights, namely, Ireland, Kazakhstan, the United States, Mexico, and Indonesia, which have weights of 0.038, 0.002, 0.764, 0.080, and 0.116, respectively. The true values of the predictors in the Table 5 are very close to the fitted values, and the fitting effect is good.

**Table 5:** Weight distribution of countries in synthetic control group (energy consumption per unit GDP).

Weight distribution of countries in synthetic control group					
Country	Irish	Kazakhstan	US	Mexico	Indonisia
Weight	0.038	0.002	0.764	0.080	0.116

Based on the weights of the countries mentioned above, China under the counterfactual framework is obtained. It can be seen that the difference between the true value and the composite value is very small, and the root mean square percentage error is only 0.081. Both of these results indicate that the fitted China well captures the characteristics of the actual China. Thus, the synthetic China can better fit the change path of energy consumption per unit of GDP and obtain a higher-precision estimate (as shown in Table 6).

**Table 6:** Descriptive analysis of prediction variable group (energy consumption per unit GDP).

prediction variable	Actual value	Synthetic value
*LNURB*	19.935	18.767
*LNGDP*	27.858	29.327
*SER*	8.058	8.074
*INV*	4.051	2.577

After fitting, the output result of Stata is shown in the figure. The solid line represents the change path of real China’s energy consumption per unit of GDP, and the dotted line represents the change path of China’s energy consumption per unit of GDP under the counterfactual framework after weighting by the control group. The degree of overlap in the previous period is relatively high. After 2006, the two lines separated rapidly, and the actual energy consumption per unit of GDP in China was less than that of China under the counterfactual framework, and this impact was significant in the long term, rather than a temporary impact.

**Figure 1:**
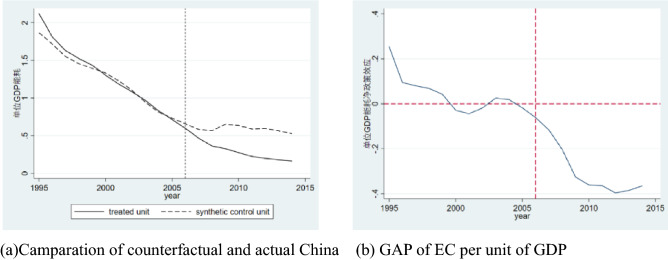
energy consumption per unit of GDP estimated by SCM.

The Fig. 1a on the right visually shows the degree of decrease in energy consumption per unit of GDP after the policy was implemented. Energy consumption per unit of GDP has always been on a downward trend in Fig. 1b. In 2010, China’s actual energy consumption per unit of GDP was 0.276, and the fitting result was 0.637, indicating that the adjustment of ECER policies reduced China’s energy consumption per unit of GDP by 0.361. Hypothesis 1 is significantly proven.

(2) Carbon emission intensity

The analysis of carbon emission intensity is as follows: Among the weights of the control group, there are four countries with non-zero weights, namely, Finland, Kazakhstan, Luxembourg and South Africa; their weights are 0.071, 0.578, 0.049 and 0.302, respectively. The sum of the values adds up to 1 (as shown in Table 7).

**Table 7:** Weight distribution of countries in synthetic control group (Carbon emission intensity).

Weight distribution of countries in synthetic control group				
Country	Finland	Kazakhstan	Luxembourg	South Africa
Weight	0.071	0.578	0.049	0.302

**Figure 2:**
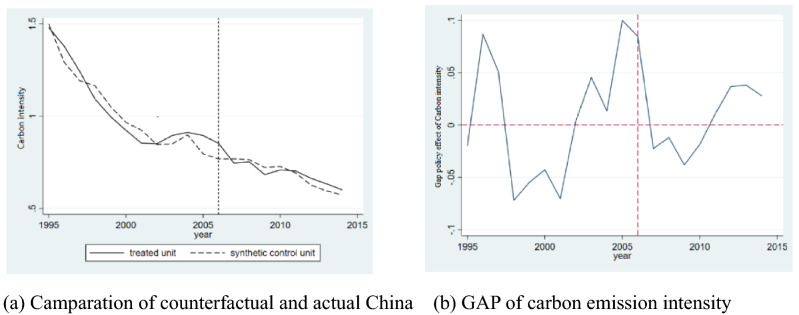
Carbon emission intensity estimated by SCM.

Based on the above weights, the RMSPE value is 0.059, but as shown in the figure, there is no obvious separation after the policy time point, indicating that the implementation of the policy has not greatly affected the carbon emission effect. The reason for this result is that the actual change trend of carbon emission intensity is not stable (as shown by the solid line in the Fig. 2), which makes the effect of synthetic control poor.

Observing the solid line in the graph, it is found that carbon emission intensity did not inherit the previous downward trend in 2003 but increased and then gradually declined. After joining the WTO, China’s manufacturing industry was more attractive, and technological innovation was less attractive. As a result, carbon emission intensity has shown an upward trend, but this finding does not mean that the policy is ineffective.

## Robustness tests

### Sensitivity analysis

**Figure 3:**
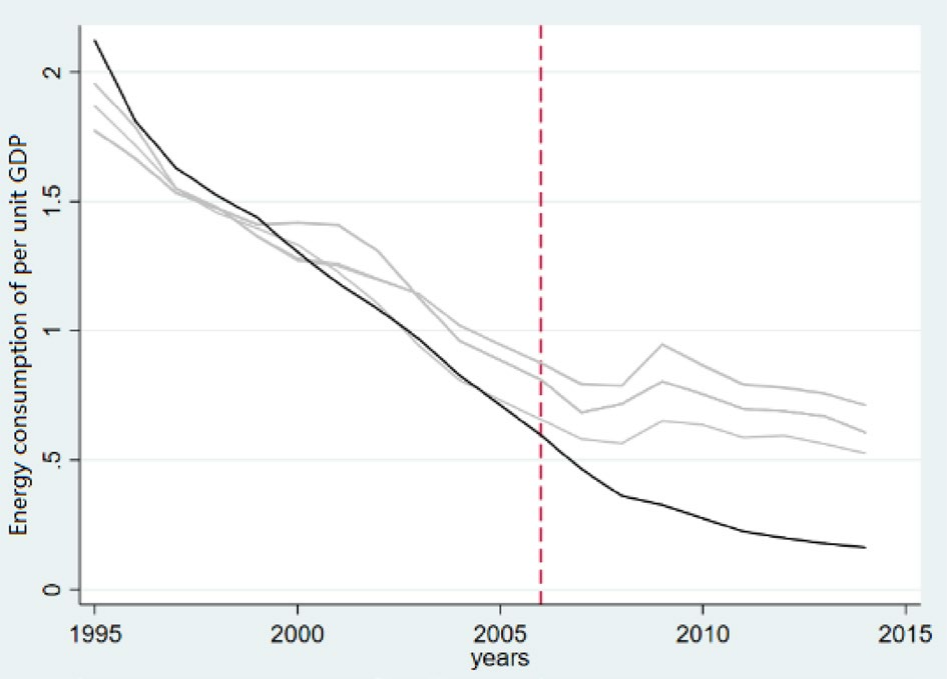
Sensitivity test of energy consumption per unit GDP.

This paper conducts a sensitivity analysis of the reduction in energy consumption per unit of GDP brought about by policies. In the figure, the solid line indicates that Ireland (0.038), Kazakhstan (0.002), the U.S. (0.764), Mexico (0.080), and Indonesia (0.116) are combined to implement energy saving and reduction. For ranked China, in the sensitivity analysis, a control group is eliminated in the order of weight from largest to smallest. The result of each iteration is represented by the light grey line in the figure, and the solid line represents the trend of unobserved China. We see that the solid line in Fig. 3 is still lower than the result of iterative synthesis, and the estimation result of the synthetic control method has not changed. This result excludes the possibility that the decrease in energy consumption per unit of GDP is affected by other policies, indicating the change in policy adjustment methods that was brought about. The effect is not affected by the individual characteristics of the control group.

### Permutation test

Before 2006, the mean square prediction error indicated the degree of fit with the actual Chinese characteristics, so the smaller the earlier mean square prediction error is, the better the degree of fit. After the policy intervention in 2006, the mean square prediction error indicated that it was affected by the policy. Regarding the degree of intervention, the greater the mean square prediction error is, the greater the impact of the policy. Therefore, the larger the ratio of the mean square prediction error between the post-policy and pre-policy is, the more significant the policy impact. Thus, we can test the significance of the impact of ECER policies by calculating the ratio of the mean square prediction error after 2006 and before 2006.

**Figure 4:**
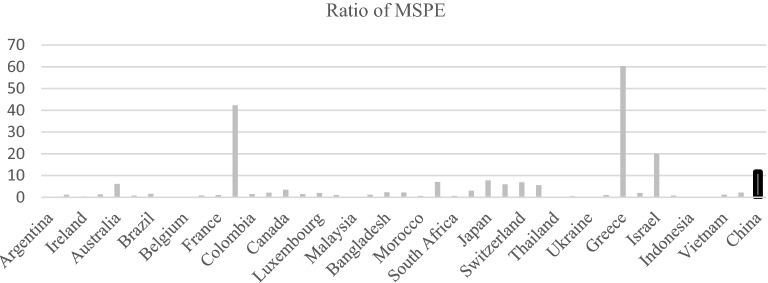
Permutation test of energy consumption per unit GDP.

During the policy window period, Denmark, the Netherlands, and Italy in the control group also implemented energy saving and emission reduction policies. To avoid their impact, after excluding these three countries, the results of the ranking test are shown in the Fig. 4. Through calculations, we find that the ratio of the mean square prediction error before and after China’s policy intervention is 11.778, which is higher than that of countries such as Finland, Greece and Israel. The distribution difference of the permutation test indicates that if one wants to achieve the same value as China, the probability of the mean square prediction error ratio is 4/43 (9.30%), indicating that the change in policy adjustment methods can be rejected at the 10% significance level. There is no null hypothesis of impacts on China’s energy consumption per unit of GDP.

### Mechanism analysis

In summary, this article argues that China has a significant inhibitory effect on ECER after changing its policy methods. Furthermore, this article analyses its impact mechanism based on previous research^19,52–54^. These studies indicate that the energy structure has an impact on energy consumption and carbon emissions. Thus, the thermal power generation rate is used to characterize the energy structure, and it is discussed as an intermediary variable.

According to Wen et al. (2004) and He et al. (2019)^55,56^, to test the mediating effect, the first step is to regress energy consumption per unit of GDP and carbon emission intensity to test whether the energy saving and emission reduction policies affect it. If the result is significant, the second step is to test whether the policy has a significant impact on the energy structure. If the coefficient of the interaction term is significant, it indicates that the policy has a significant impact on the energy structure of the intermediary variable, and carbon emission intensity is regressed. If the effect of the intermediary variable is significant and the coefficient of the interaction term decreases or is not significant, this result means that the policy affects energy consumption per unit of GDP and carbon emission intensity by affecting the energy structure.

The regression results are shown in Table 8. The coefficients of the effect of the thermal power generation rate on energy consumption per unit of GDP and carbon emission intensity are 0.075 and 0.005, respectively, and both are positively correlated, indicating that increased use of coal will increase ECER and that the mediating effect accounts for 0.172 and 0.540, respectively. The results show that Hypothesis 3 is true: ECER accountability can drive local officials to achieve policy goals by increasing the clean power generation rate.

**Table 8:** Test results of mediation effect.

Variable	Energy consumption per unit of GDP	Carbon emission intensity
	Model (5)	Model (6)
The energy structure	0.075*** (0.017)	0.005*** (0.068)
Control variables	Controlled	Controlled
ECER*YEAR	Controlled	Controlled
Cons	984.848*** (160.116)	26.691*** (2.414)
R^2^	0.559	0.487
SOBEL Z	3.158	4.405
SOBEL Z-P value	0.002	0.000
GOODMAN-1 Z	3.119	4.400
GOODMAN-1 Z-P value	0.002	0.000
GOODMAN-2 Z	3.198	4.409
GOODMAN-2 Z-P value	0.001	0.000
Mediation effect	3.029	0.216
Proportion of mediating effect	0.172	0.540

In parentheses are standard errors, *, ** and *** indicate significance levels of 1%, 5% and 10%, respectively.

## Discussion

In the empirical analysis, two counterfactual estimation methods, the DID method and the SCM, are used to identify the environmental accountability effect of ECER incorporated into the objectives of the five-year plan. The research shows that by including the five-year plan from 2006 into the ECER action objectives, the implementation of the official accountability system has strengthened the existing environmental protection restraint effect, formed a major incentive for actual ECER work under the existing environmental protection legislation and administrative measures, promoted the general trend of a reduction in energy consumption per unit of output value in China's economic development, and attenuated carbon dioxide emission intensity in the long term.

### Emission target accountability of five-year plan and reduction of energy consumption per unit of output value

The DID method and the SCM are used to verify the impact of ECER target accountability on the decline in actual energy consumption intensity. Different estimation methods show the same conclusion. China's ECER target responsibility system makes the country’s energy consumption intensity significantly lower than that of the other 45 countries. This decline is more prominent than the counterfactual China formed by other control groups in the comparative study of countries, as shown in Fig. 1b. Among the countries that have signed the Kyoto Protocol and the Paris Climate Agreement, many governments have carried out environmental legislation and announced phased ECER objectives^26,29^, but only China has effectively assessed the environmental performance of officials through ECER target accountability^33^. China's local governments have a strong administrative capacity. Under the performance evaluation path of the "promotion tournament", major officials have a strong motivation to meet standards by using environmental governance ability^5,42^, and governments can also bear the enormous economic costs for ECER.

First, local ECER is incorporated into the target responsibility system of the five-year plan, which has a significant "demonstration effect". The regions that take the lead in large-scale energy conservation efforts have excellent performance evaluations and promotions of officials, which plays a strong exemplary role in other regions that fail to meet the energy conservation goal^57^. Enterprises with high energy consumption and high emissions will gradually move out of areas with more stringent ECER requirements due to policy competition and pour into areas with weak environmental constraints. They will also reduce their energy consumption and carbon emissions through technological transformation to obtain rewards from the government^28^.

Second, under the existing technical conditions, there are short-term technical obstacles to reducing the carbon emission intensity per unit energy consumption. However, reducing energy consumption in economic activities and, thus, reducing total emissions are a suboptimal choice, which could work in the short term and can be economically borne by the government^58^. In relatively developed regions, environmental governance and energy consumption emissions have higher social voices and stronger transparency requirements for environmental emission supervision^33^. Local regimes have both the popular support and the economic clout to keep energy consumption down, and they can afford not to pursue the absolute size of economic output.

### The target responsibility system written into the five-year plan and the decline of carbon emission intensity

The study shows the policy effect of carbon emission intensity through Table 4, Models (3) and (4) and Fig. 2. Different estimation methods obtain different conclusions. Models (3) and (4) based on DID estimation show that the carbon emission intensity per unit of GDP decreases significantly, but the graphical analysis of the SCM shows no significant results. The reason is that the carbon emission intensity of the unit output value is subject to the economic development level of each region and the level of use of advanced technology for energy conservation and emission reduction. In China, there are large regional differences in the economic development level and regional differences in unit energy consumption^59^. In addition, the energy factor endowment level between western energy-exporting provinces and eastern energy-importing provinces differs greatly, and there are regional differences in terms of energy prices. The common trends in DID estimation could not identify differences in the unobserved variables in China, but the SCM could indirectly synthesize China without ECER accountability through country comparison. Therefore, the research takes the SCM as an improvement on DID estimation.

In addition, for local governments, policy-based emission reduction and power rationing to reduce production are aggregate control methods with obvious short-term effects, which are applicable to energy intensity. However, reducing carbon emission intensity is a long-term process of technology application and diffusion in which policy implementation takes a long time, and the effect is not easy to detect. As reflected in this study, the effect of energy saving is significant, but the effect of emission reduction is insufficient. Therefore, the long-term goal of carbon peaking can be achieved only through the unremitting promotion and popularization of emission reduction technologies and the gradual development of clean production. China cannot be better than other countries in terms of technology and economic stimulus, while other countries around the world actively put into practice the Kyoto Protocol and Paris Climate Agreement to attain emission reduction targets simultaneously. Nevertheless, ECER target accountability system improvement could reinforce ECER regulation in China, bringing a unique advantage that would be hard for others to obtain.

### Accountability of officials to achieve ECER objectives decrease the proportion of coal and electricity

After China's accession to the WTO, trade liberalization objectively exacerbated the overall rise of the country's carbon emission level^60^. FDI triggered the migration of high-carbon production links from developed countries to China, making China the world factory. Over the years, FDI has formed a point-to-area industrial agglomeration belt with high energy consumption and high carbon emissions, posing a direct challenge for local regimes to achieve the goal of ECER. To balance economic development and ECER goals, China has promoted an increase in the proportion of clean power generation in the power generation energy mix and substantially has alleviated the situation of high carbon emission intensity by implementing the ECER target accountability system for local officials^61^ and comprehensively addressing the problem of burning too much coal in domestic socio-economic production. On the other hand, a large amount of fossil energy is used for power generation; as long as the effective proportion of coal-fired units in the power generation energy structure is reduced, the situation of high carbon emission intensity can be greatly alleviated^62^.

To fulfil ECER's stipulated goals, local governments arrange state-owned power generation enterprises to reduce the proportion of coal power and carry out continuous adjustment and optimization of the clean power generation structure^40^. At the beginning of the Eleventh Five-Year Plan period, coal-fired power generation accounted for 88% of China's primary energy structure. Through continuous efforts to transform the stock of coal-fired power and introduce incremental clean power generation, the proportion of coal-fired power has been reduced to slightly under 65% in 2020. Local power generation enterprises are SOEs or administratively binding enterprises that are closely related to regimes. In the process of performing energy-saving tasks, local officials have sufficient ability to mobilize power generation enterprises to actively adjust the power generation structure and not only consider current profits. The accountability system for ECER drives local officials to actively carry out the practice of institutional innovation through the carbon emission trading of power generation enterprises and clean power generation trading in the green card market to accelerate the decrease in the coal power share of power generation enterprises.

## Conclusion and policy suggestions

This article studies the changes in China’s ECER after the target accountability system was implemented in local regimes. In the practice of ECER, China has shifted its new approach by implementing the ECER accountability of local regimes as an enhancing institutional mechanism. The paper observes the special method for strengthening the effect of existing ECER policies and employs DID and SCM models to estimate the policy effect of ECER accountability implementation. The research results show that changes in environmental regulation and policy adjustment have a depressing effect on energy consumption and emissions. The government has also incorporated energy conservation and emission reduction targets into its five-year plans. Therefore, emerging forms of ECER policies can be applied with local regimes’ responsibility. Bureaucracies are always paying attention to the promotion indicators listed by the central government. Their intention of promotion would facilitate the achievement of the ECER target under the guidance of accountability.

This paper finds that the implementation of ECER policies strongly depends on the alignment of targets set by the accountability baton of each local regime. As economic development is the primary goal, local officials will pursue increases in GDP; when ECER indicators are included in the assessment of the target accountability system, the incentive for officials to implement emission reduction responsibility is more significant. This performance tournament features the following suggestions for further promoting ECER work:

a) Authoritarian governments can decentralize ECER targets to incentivize local officials to fulfil their responsibilities rather than frequently promulgating restrictive rules and decrees. By driving officials' performance target orientation, all departments are encouraged to implement existing ECER measures and tighten restrictions to achieve the goals of ECER. Through long-term practice, China has found medium- and long-term effects of the ECER target responsibility system. Therefore, in 2015, the mayor responsibility system was upgraded to the party secretary responsibility system, which further strengthened the incentive role of local officials in the implementation of ECER. Only when the local bureaucratic system can keep its promotions consistent with the ECER goals can the positive externality effect of ECER be internalized by institutional innovation, forming political incentives to optimize methods to facilitate the achievement of ECER targets.

b) The focus of promoting a further deepening of ECER in the next stage is to realize the transformation of the energy consumption structure and promote the effective replacement of the stock of traditional fossil energy with incremental clean energy. The total energy consumption level and unit energy intensity level are subject to the technical conditions in a certain period, and their decreasing trend is weaker than that of the total energy consumption level and emission intensity. Therefore, the focus of further ECER should be on reducing emissions rather than reducing total energy consumption. Many countries have seen renewable energy generation as a major opportunity to drive the carbon decoupling of economic activity. In China, clean generation is replacing the coal-fired power generation stock at a rate of approximately 1% per year. In the face of the current ratio of nearly 65% coal-fired power generation, there is still a gap between achieving a carbon peak in 2030 and carbon neutrality in 2060, and the speed of clean power generation replacement needs to be significantly increased.

c) There is convergence in the setting of the periodic indicators of the ECER target accountability system. Both the overall and intensity indicators need to be adjusted based on the actual emission reduction of local conditions. To ensure the achievement of ECER targets, local officials have resorted to power rationing to avoid reaching the upper limit of annual carbon emission targets, which affects local economic development and people’s livelihoods. To avert overcorrection, local accountability systems need to set scientific and reasonable phased targets for ECER and reduce the substantial damage to the sustainability of economic growth caused by over-aggressive targets.

## Data Availability

All data used for analysis for this study are in public domain and available free of charge at https://www.bp.com/en/global/corporate/energy-economics/statistical-review-of-world-energy/downloads.html, and https://databank.worldbank.org/source/world-development-indicators.
